# Adolescents’ Knowledge of Breastfeeding and Their Intention to Breastfeed in the Future

**DOI:** 10.3390/children4060051

**Published:** 2017-06-20

**Authors:** Marija Čatipović, Tamara Voskresensky Baričić, Sunčana Rokvić, Josip Grgurić

**Affiliations:** 1 General Pediatric Office Marija Čatipović, 43000 Bjelovar, Croatia; 2 General Pediatric Office, Community Health Centre Zagreb-Centar, 10000 Zagreb, Croatia; baricict@gmail.com; 3 Žiraha services Ltd, 10000 Zagreb, Croatia; suncanavujic@gmail.com; 4 UNICEF Office for Croatia, 10000 Zagreb, Croatia; jgrguric@unicef.hr

**Keywords:** breastfeeding, school children education

## Abstract

The aim of this paper is to analyze third-year secondary school students’ knowledge of breastfeeding and intention to breastfeed their children, based on the results of a questionnaire. The respondents were 154 students (101 female/43 male) of two secondary schools in Bjelovar. The students completed a questionnaire which consisted of 23 questions regarding knowledge and intention to breastfeed. The answers were analyzed statistically and different results were compared by nonparametric tests. About half of the respondents think that both partners should decide on breastfeeding and recognize the role that fathers have in initiating and maintaining breastfeeding. Only 13.64% of the respondents know that breastfeeding is to be done only on demand. Exclusive breastfeeding for 6 months, as recommended by the medical profession, is recognized by 70.13% of the students. The question on how justified is the initiation of formula together with the mother’s milk was answered correctly by 29.22% of the students. Secondary school students’ knowledge of breastfeeding is insufficient, and schools, families, social communities and other sources of information should share the responsibility for improving this. We consider it necessary to pay more attention to improving students’ knowledge of breastfeeding through school curricula.

## 1. Introduction

The UNICEF programme, the Baby-Friendly Hospital Initiative, was launched in Croatia in 1993 [1]. Today, all Croatia’s maternity wards are baby-friendly. The further goal is to improve the care for mothers in these maternity wards in order to make them mother-friendly (the Mother-Friendly Hospital Initiative). The programme Community Supporting Breastfeeding in Croatia has been based on the cooperation between medical workers and various segments of society to promote and support breastfeeding; it is aimed at five groups: the programme team staff; the health system; partners from various segments of society; the media; breastfeeding mothers; pregnant women and their families. A Croatian programme which takes the specific qualities of our health system into consideration has also been developed under the name Counselling for Children-Breastfeeding-Friendly. In spite of all the efforts, the results are not satisfactory [2,3,4]. However, it should be mentioned that we still do not have a well-organized, structured and systematic data set on breastfeeding [5].

It goes without saying that breastfeeding is the best nourishment for a child [6,7,8]. The question is how to increase the rate of breastfeeding. Different factors that may influence the frequency of breastfeeding in a local community have been considered [9,10,11]. However, there are no papers that deal with the factor of young people’s knowledge and attitudes concerning the presence of breastfeeding in a certain population in Croatia. We believe that activities directed towards adolescents and younger children have been neglected, which is important because attitudes on breastfeeding are formed in the early phase of adolescence [12,13].

The aim of this study is to analyze knowledge and intentions related to breastfeeding among the third-year students of School of Economics and Business Administration Bjelovar and Nursing School Bjelovar.

## 2. Methods

The study was conducted in Bjelovar in March, April and May 2016. In total, 154 students participated in the survey, 85 from Nursing School Bjelovar and 69 from School of Economics and Business Administration Bjelovar. Third-year students were chosen because they have completed a compulsory, unique, educational programme that is carried out during the first two years of secondary education. While planning this study, Ethical Standards for Research with Children [14] were respected and, before the beginning of the study, the approval of the schools’ Ethical boards was acquired which means that the study was conducted in compliance with the ethical principles and professional standards valid in our country.

Despite the fact that the respondents were students of two different secondary schools, they had similar socio-demographic characteristics; there were no significant differences in relation to their age, sex, place of residence, their parents’ educational qualifications, and the experience of being/not being breastfed in childhood. (Table 1). There were significant differences only in their success at school (*p* = 0.0147).

Contact was made with the schools by informing the schools’ headmasters about the intention and the purpose of the study. A letter with a copy of the questionnaire was sent to the schools whose headmasters were willing to cooperate, and approval for conducting the study in their school was requested from the schools’ leadership. There were no preparatory lectures for this questionnaire in the schools; there were no lectures on the subject of breastfeeding outside the regular school curriculum. The students were previously informed about the content and the purpose of this questionnaire, about the information they would need to obtain from their parents, and when the questioning will take place. The students were not obliged to take part in the study and everyone who arrived at the arranged time was able to fill in the questionnaire. The students were not in any way rewarded for taking part in the study, nor was their arrival in any way noted. The students completed the questionnaires on computers. The completion of the questionnaire was supervised by the schools’ professional staff and the participants of the study made no contact with the other students. While logging in to a computer, the students gave no personal information or any other kind of data that could identify the person who completed the questionnaire.

The data was gathered using a questionnaire (Supplementary Material 1). The questionnaire was made according to a sample of The Iowa Infant Feeding Attitude Scale. [15]. The questionnaire was not validated. As far as the formation of questions is concerned, there was a version for male respondents and a version for female respondents. The questions about breastfeeding in the version for male respondents were formulated regarding their support of the female partner’s decision (Supplementary Material 2). The questionnaire consists of three parts. The first part includes general and social information (age, sex, success at school, place of residence, educational qualification of the parents, and the experience of being breastfed in childhood). The second part deals with the intention to breastfeed (establishing breastfeeding after the delivery, breastfeeding when a father does not support it, breastfeeding in public, joint decision making on breastfeeding, breastfeeding after returning to work, breastfeeding after the child’s first and second year of life, breastfeeding recommended by doctors regardless of the family’s attitude). The third part includes knowledge of breastfeeding (the contents of mother’s milk, the advantages of mother’s milk, exclusive breastfeeding, father’s support, medication and breastfeeding, milk formula). The answers to the questions regarding the intention to breastfeed are graded from 1 to 5, 1 and 5 denoting total acceptance and total unacceptance (“I totally agree/disagree.”) of an intention, respectively. The answers 2 and 4 present acceptance and unacceptance, respectively, while the answer 3 encompasses a group of indecisive students (“I do not know.”). “Correct” or “incorrect” are the possible results of students’ answers to the questions which test the students’ knowledge of the subject.

The results were analyzed using Microsoft Excel, Microsoft Office 2010. The comparison of the results was based on the students’ sex and whether they were or were not breastfed in their childhood. The testing of the differences between the groups was performed by using the X^2^ test and Yates correction was used where necessary. The level of significance *p* ≤ 0.05 was used [16,17].

## 3. Results

Table 2 shows the respondents’ answers regarding the intention to breastfeed. In total, 2.6% of the respondents would not try to establish breastfeeding after the delivery and would immediately feed the baby with substitute milk formula. There is no significant difference between the answers of breastfed children and the answers from children who were not breastfed (*p* = 0.867), or between the sexes (*p* = 0.867).

The majority of respondents (83.77%) confirm their intention to breastfeed even if their partner does not support the decision. The differences between the students’ answers are not statistically significant regarding their sex and their experience of being/not being breastfed in childhood.

In total, 47.40% of the respondents stated that they would not breastfeed in public, for example, in a restaurant or a park. The difference between the answers regarding the intention to breastfeed in public is not significant regarding the students’ sex, or their experience of being/not being breastfed in childhood.

In total, 26.63% of the respondents disagree (“I totally disagree” and “I disagree”) with the statement that both parents should make a decision on breastfeeding. The difference between the answers of breastfed children and the ones who were not breastfed is not significant, but there is a statistically significant difference in relation to the respondents’ sex. A significantly larger number of male respondents accept the intention to make a joint decision on breastfeeding (*p* = 0.000).

In total, 62.34% of the respondents “agree” and “totally agree” that returning to work would not stop them from breastfeeding. There is no significant difference between the breastfed children and the ones who were not, but there is a significant difference between the sexes. A significantly larger number of female respondents stated this intention (*p* = 0.002).

In total, 43.51% of the students questioned declared that it is acceptable (“accept” and “totally accept”) to continue breastfeeding after the child’s first year of life. There is no statistically significant difference between the answers of different sexes or between participants who were or were not breastfed. Only 9.74% of the participants disagree (“totally disagree” and “disagree”) with the cessation of breastfeeding after the child’s second year of life.

In total, 75.97% of the respondents “accept” or “totally accept” breastfeeding a child in accordance with doctors’ recommendations, regardless of the family’s attitude. The respondents’ answers do not statistically differ in regards of their sex and their experience of being breastfed or not.

Table 3 shows the respondents’ answers to the questions about knowledge of breastfeeding. There are commentaries in the text on the questions with less than 50% correct answers, or where there is a determined, statistically significant difference between the respondents of different sexes and the respondents’ experience of being breastfed in childhood. Only 21 respondents knew that breastfeeding should be done on demand (16.28% of male respondents and 12.61% of female respondents answered correctly). In total, 45 students gave a correct answer that mother’s milk is always of sufficient quality and that there is no need to introduce milk formula while breastfeeding (37.21% of male respondents and 26.13% of female respondents). The majority of the respondents (144) knew that breastfeeding is useful for establishing emotional attachment (13.95% of male respondents and 3.6% of female respondents answered incorrectly; *p* = 0.048). Only 37 respondents knew that taking any kind of medication is not a reason to stop breastfeeding (25.58% of male respondents and 23.42% of female respondents answered correctly). Only 71 students recognized the significant role of the father in initiating and maintaining breastfeeding (60.74% of male respondents and 40.54% of female respondents; *p* = 0.040). The intention to stop breastfeeding after a child turns two shows a significant negative correlation with the opinion that breastfeeding is not necessary when a child starts eating baby porridge (*r* = −0.201, *p* = 0.012). Only 65 students knew that it is not necessary to give water to a child who is breastfed (58.14% of male and 57.66% of female respondents answered incorrectly).

## 4. Discussion and Conclusions

This study demonstrates that a great number of female and male respondents support the intention to establish breastfeeding at a maternity ward, right after childbirth; less than a half find it acceptable to breastfeed for more than a year; and approximately 10% find it acceptable to breastfeed for more than two years. A much higher percentage of female respondents, as compared to the percentage of male respondents, think that returning to work would not stop them from breastfeeding, and a higher percentage of male respondents think that a child’s mother and father should both make a decision on breastfeeding. A great percentage of respondents (around 80%) support the intention to breastfeed even if the father does not support it, as well as breastfeeding according to doctors’ recommendations, regardless of other family members’ opinions. The respondents did not show sufficient knowledge of breastfeeding, especially of the influence of medication on breastfeeding, the importance of breastfeeding on demand, the justifiability of introducing milk formula alongside breastfeeding, the need to give water alongside mother’s milk, etc. When comparing female and male respondents’ answers, male respondents had a significantly larger number of correct answers to the question about the importance of the father’s support during the establishment of breastfeeding and female respondents had more correct answers to the question about the influence of breastfeeding on developing emotional attachment.

The percentage of respondents who, already at their adolescent age, show no intention to try to breastfeed at a maternity ward is concerning because the intention to behave in a certain way is an important link between an attitude and behavior [18], and strongly predicts future behavior [19]. The fact that mothers know that mother’s milk is extremely important, especially for newborn children [20], does not mean that they will breastfeed. Without knowing information about the specific reductions in health risks that occur through breastfeeding and the consumption of breast milk [21], mothers cannot properly weigh the advantages and disadvantages of breastfeeding versus formula feeding, and thus they cannot make a truly informed decision about how they want to feed their babies. In Croatia, the percentage of children who are breastfed right after leaving a maternity ward is 91–95 percent [22]. If we want to increase this percentage, we have to influence the younger population because many children and adolescents have already considered infant feeding choices for when they become parents [23,24,25].

The decision to breastfeed has a considerable effect on how a woman functions in her family and at work [26], and has a significant effect on marital relations [27]. Many women mistakenly think that they cannot breastfeed if they plan to return to work after childbirth, and thus they may not talk with their employers about their desire to breastfeed or how breastfeeding might be supported in the workplace [28]. Numerous factors influence the duration of breastfeeding [29]; a male partner’s negative attitude towards breastfeeding can be an obstacle for initiating and maintaining breastfeeding, and vice versa [30,31]. Studies show that breastfeeding is much more successful and long-lasting in families where both the father and the mother have positive attitudes towards it [32]. The partner and the baby’s grandmothers play critical support roles when it comes to breastfeeding, both with regard to assisting in decision making about how the baby is fed and in providing support for breastfeeding after the baby is born [33,34,35,36]. Therefore, we need to strengthen social awareness of the importance of parents’ joint decision-making concerning breastfeeding and the whole family’s support of the mother’s decision to breastfeed.

Mothers who nurse longer than the social norm sometimes hide their practices from all but very close family members and friends; this is called “closet nursing” [37]. In our study, the percentage of respondents who find it acceptable to breastfeed after the child’s first year of life is slightly bigger than the percentage of mothers who breastfeed for more than a year in our surroundings [38]. The guidelines of UNICEF, ESPGHAN Nutrition Committee, Croatian Society of Pediatric Gastroenterology, Hepatology and Nutrition support breastfeeding even after the child’s first year of life “until it suits the mother and her child” [39,40]. Elizabeth Baldwin says: “Because our culture tends to view the breast as sexual, it can be hard for people to realize that breastfeeding is the natural way to nurture children” [41]. Only 27.92% of the respondents disagreed with the statement that they would not breastfeed in a restaurant or a park, which could be conditioned by social norms [42,43]. In many countries, the right to breastfeed in public has been established by law. Law makers have acknowledged that the right to food is a fundamental human right [44]. Discomfort with the idea of breastfeeding in public has been cited as a reason for some women choosing not to initiate breastfeeding [45] or planning a shorter duration of breastfeeding [46]. Groleau and colleagues point to “the urgent need for reintroducing the nutritional role of the breast into various social and public spaces including the medias” [47].

We have taken measures to ensure mothers and their children have adequate places for breastfeeding in public (“Drinking coffee with mothers” and “Places for breastfeeding in the means of public transport”) [48].

The research works of other authors confirm the need and the possibility to educate primary and secondary school students about breastfeeding [49,50,51,52], as well as further research on the subject [53], because there is a proven connection between positive attitudes towards breastfeeding and successful breastfeeding in later life [54]. The World Health Organization endorses strategies and programs that work with children and adolescents to provide education that promotes awareness and positive attitudes towards breastfeeding as part of the general curriculum [55]. The UNICEF Baby Friendly Initiative also recommends breastfeeding education in schools [56].

There are several limitations to the present study. The first limitation is the relatively small number of respondents, which is due to the very complex procedure of acquiring the approval of the Ethical boards and the School’s councils for conducting the study at their school. The second limitation is connected to the first one because the questionnaire used in the study is not validated; validation requires a large number of respondents. The third limitation is that the questionnaire does not offer the possibility to analyze the reasons behind certain answers.

In summary, despite the limitations, we believe that the study allows us to draw the conclusion that it is necessary to work with the respondents in order to develop positive attitudes and improve their knowledge of breastfeeding, especially regarding the important questions of initiating breastfeeding in maternity wards, exclusive breastfeeding for six months, the father’s support of breastfeeding, breastfeeding for as long as the mother and the child desire, etc. Schools are ideal places for carrying out such education [57,58,59].

## Figures and Tables

**Table 1:** Socio-demographic characteristics of the respondents.

	Nursing School Bjelovar	School of Economics and Business Administration Bjelovar	Total
**Sex**			
Female	64	47	111
Male	21	22	43
**Average age**	17.247	17.072	17.170
**Place of residence**			
Village	53	33	86
City/town	32	36	68
**Mother’s educational qualifications**			
Primary/secondary education	69	56	125
Two-year/university degree	16	13	29
**Father’s educational qualifications**			
Primary/secondary education	74	57	131
Two-year/university degree	11	12	23
**School grade average**			
Good	9	20	29
Very good	53	34	87
Excellent	23	15	38
**Breastfed in childhood**			
Yes	74	63	137
No	11	6	17

**Table 2:** The answers to the questions about breastfeeding intent.

Intentions *	Male (*n*)					Female (*n*)					
	5	4	3	2	1	5	4	3	2	1	*p*
After the delivery, I would not try to establish breastfeeding. I would bottle-feed my child with formula milk.	0	1	4	13	25	0	3	5	18	85	/
I would breastfeed my child even if the child’s father does not support my decision to breastfeed.	17	19	5	1	1	67	26	10	6	2	/
I would not breastfeed in public, for example in a restaurant or in a park.	9	11	9	8	6	27	26	29	19	10	/
A child’s mother and father should make a joint decision about breastfeeding.	17	15	7	3	1	11	30	33	20	17	0.000
Returning to work would not make me stop breastfeeding.	9	9	23	1	1	40	38	25	5	3	0.002
I find it acceptable to breastfeed after a child turns one if a child so desires.	9	11	11	9	3	17	30	27	24	13	/
I would breastfeed my child in accordance with doctors’ recommendations, regardless of close family members’ opinions.	19	18	5	0	1	38	42	21	8	2	/
I would not breastfeed my child after he/she turns two.	12	13	13	2	3	44	30	27	6	4	/

* Legend: 5 = I totally agree; 4 = I agree; 3 = I have no opinion; 2 = I disagree; 1 = I totally disagree.

**Table 3:** The answers to the questions about knowledge of breastfeeding.

Questions about knowledge of breastfeeding	Male (*n*)		Female (*n*)		*p*
	Correct	Incorrect	Correct	Incorrect	
Mother’s milk is poor in iron.	40	3	106	5	
A child needs to be breastfed on schedule, every three to four hours.	7	36	14	97	
Medicinal experts recommend exclusive breastfeeding (without adding water or solid food) until a baby is 6 months old.	30	13	78	33	
Breastfeeding protects a child from infectious diseases and allergies.	42	1	101	10	
Breastfeeding accelerates children’s brain development.	36	7	91	20	
Education on breastfeeding should start during a mother's pregnancy.	37	6	84	27	
Mother’s milk is not sometimes of a sufficient quality so it is necessary to introduce formula feeding alongside breastfeeding.	16	27	29	82	
Breastfeeding has proved to be useful for developing emotional attachment between a mother and a child.	37	6	107	4	0.048
If a mother is ill and takes medication, it is necessary to stop breastfeeding.	11	32	26	85	
If the child is fed by formula milk in the maternity ward, it is not possible to establish successful breastfeeding at home.	41	2	100	11	
The substitute formula milk is equally nutritious and of the same quality as mother’s milk.	39	4	103	8	
A father’s support, such as his presence during the delivery, facilitates the establishment of breastfeeding.	26	17	45	66	0.040
When a child is able to start eating baby porridge, breastfeeding is not necessary.	26	17	54	57	
A child needs to be given water alongside breastfeeding from birth.	18	25	47	64	
Breastfeeding has a positive impact on a child’s health later in life.	41	2	108	3	

## Supplementary Material

The following are available online at www.mdpi.com/2227-9067/4/6/51/s1, Supplementary S1: Female Questionnaire, Supplementary S2: Male Questionnaire.
