# Supplementary material for: Adolescents’ Knowledge of Breastfeeding and Their Intention to Breastfeed in the Future
Source: Children (Basel). 2017 Jun 20;4(6):51. doi: 10.3390/children4060051 (PMC5483626; doi:10.3390/children4060051)
Supplement: Supplementary file 1 [file children-04-00051-s001.pdf]

Supplementary Material:

Supplementary 1. Female Questionnaire.

The questionnaire:

- How old are you? ____
- Where have you spent most of your life?

  in a village

  in a town

  in a large town or a city
- Which grade average did you achieve in the previous grade of secondary school?

  insufficient

  sufficient

  good

  very good

  excellent
- What are your mother's and your father's educational qualifications?

  Mother: a) primary education

  b) secondary education

  c) two-year degree

  d) university degree

  e) master's degree

  f) doctor's degree

Father: a) primary education

b) secondary education

c) two-year degree

d) university degree

e) master's degree

f) doctor's degree

- Were you breastfed as a child? YES NO

  If yes, write for how many months: ________________________

- What was the reason for the cessation of breastfeeding?

  ____

- Would you like to learn more about breastfeeding at school? YES NO
- Here you can read a number of claims about breastfeeding. Please mark in which extent you agree or don't agree with each claim by circling the number which coincides with your opinion the most. There are no correct and incorrect answers. Don't think too much, circle what you think of first.

  1 = I totally disagree

  2 = I disagree

  3 = I have no opinion

  4 = I agree

  5 = I totally agree

	I totally disagree	I disagree	I have no opinion	I agree	I totally agree
1. After the delivery, I wouldn't try to establish breastfeeding. I would bottle-feed my child with formula milk.	1	2	3	4	5
2. I would breastfeed my child even if the child's father doesn't support my decision to breastfeed.	1	2	3	4	5
3. I wouldn't breastfeed in public, for example in a restaurant or in a park.	1	2	3	4	5
4. A child's mother and father should make a joint decision about breastfeeding.	1	2	3	4	5
5. Returning to work wouldn't make me stop breastfeeding.	1	2	3	4	5
6. I find it acceptable to breastfeed after the child first year of life if the child desires so.	1	2	3	4	5
7. I would breastfeed my child in consistence with the doctors' recommendations, regardless of the closer family members' opinions.	1	2	3	4	5
8. I wouldn't breastfeed my child after it turns two.	1	2	3	4	5

- Your current knowledge of breastfeeding has mostly been influenced by: (please, rank the given answers from 1 to 6, putting the number 1 next to the answer regarding who/what has had the greatest influence on you, and number 6 next to the answer regarding who/what has had the least influence on you)

  ________ school

  ________ mother

  ________ magazines

  ________ television

  ________ friend

  ________ the Internet

- Would you like to have your partner present during childbirth? YES NO

  If not, write down who would you like to be present during childbirth? ___________________

- Here you can read the questions regarding the knowledge of breastfeeding. Read each claim carefully and assess whether it is correct or incorrect. It is possible that you won't know the answers to some of the questions, but, nevertheless circle the one you consider to be correct. Please, don't copy other students' answers.

1. Mother's milk is poor in iron.	CORRECT	INCORRECT
2. A child needs to be breastfed on schedule, every three to four hours.	CORRECT	INCORRECT
3. Medicinal experts recommend exclusive breastfeeding (without adding water or solid food) until a baby is 6 months old.	CORRECT	INCORRECT
4. Breastfeeding protects a child from infectious diseases and allergies.	CORRECT	INCORRECT
5. Breastfeeding accelerates children's brain development.	CORRECT	INCORRECT
6. Education on breastfeeding should start during a mother's pregnancy.	CORRECT	INCORRECT
7. Mother's milk isn't sometimes of a sufficient quality so it is necessary to introduce formula feeding alongside breastfeeding.	CORRECT	INCORRECT
8. Breastfeeding has proved to be useful for developing emotional attachment between a mother and a child.	CORRECT	INCORRECT

9. If a mother is ill and takes medication, it is necessary to stop breastfeeding.	CORRECT	INCORRECT
10. If the child is fed by formula milk in the maternity ward, it is not possible to establish successful breastfeeding at home.	CORRECT	INCORRECT
11. The substitute formula milk is equally nutritious and of the same quality as mother's milk.	CORRECT	INCORRECT
12. A father's support, such as his presence during the delivery, facilitates the establishment of breastfeeding.	CORRECT	INCORRECT
13. When a child is able to start eating baby porridge, breastfeeding is not necessary.	CORRECT	INCORRECT
14. A child needs to be given water alongside breastfeeding from birth.	CORRECT	INCORRECT
15. Breastfeeding has a positive impact on a child's health later in life.	CORRECT	INCORRECT

Supplementary 2. Male Questionnaire.

Here you can read a number of claims about breastfeeding. Please mark in which extent you agree or don't agree with each claim by circling the number which coincides with your opinion the most. There are no correct and incorrect answers. Don't think too much, circle what you think of first.

1= I totally disagree

2 = I disagree

3 = I have no opinion

4= I agree

5 = I totally agree

	I totally disagree	I disagree	I have no opinion	I agree	I totally agree
1. I would support my partner's decision not to breastfeed after the delivery and to bottle-feed the child by substitute milk formula.	1	2	3	4	5
2. I would encourage my partner (the mother of our child) to breastfeed even if she didn't show any desire to do so.	1	2	3	4	5
3. I wouldn't support my partner's (the mother of our child's) decision to breastfeed in public, for example in a restaurant or a park.	1	2	3	4	5
4. The child's mother and father should make a joint decision on breastfeeding.	1	2	3	4	5
5. I would support my partner's (the mother of our child's) decision to continue breastfeeding after returning to work.	1	2	3	4	5
6. I find it acceptable that my partner (the mother of our child) breastfeeds our child after it turns one, if the child desires so.	1	2	3	4	5
7. I would give my partner (the mother of our child) support to breastfeed in consistence with the doctors' recommendations, regardless of the closer family members' opinions.	1	2	3	4	5
8. I wouldn't support my partner (the mother of our child) to breastfeed after the child's second year of life.	1	2	3	4	5
